# Supplementary material for: Patterns of Intron Gain and Loss in Fungi
Source: PLoS Biol. 2004 Nov 30;2(12):e422. doi: 10.1371/journal.pbio.0020422 (PMC532390; doi:10.1371/journal.pbio.0020422)
Supplement: Table S1 — Also available at http://genes.mit.edu/NielsenEtAl/. (4.3 MB ZIP). [file pbio.0020422.st001.zip › NielsenEtAl/html/1332.html]

AN5528.1.NCU05029.1.MG03406.1.FG10911.1


```
 CLUSTAL W (1.82) Multiple Sequence Alignments - Introns Inserted


Sequence 1: MG03406.1	700 aa
Sequence 2: FG10911.1	698 aa
Sequence 3: NCU05029.1	716 aa
Sequence 4: AN5528.1	701 aa
Alignment Length: 726 aa
Number Identitical Residues: 398 aa
Alignment Score (without introns) 17487


MG03406.1 	MIT-RNIPRAALLSSRPTLSSCQHRHLPPWPPRFPSSR-------IRALITSTPRR--AS
NCU05029.1	MAPSIKLSTMATSLHRAHGTSALLRRPRLWAPRLSSIHATPTIANLRASFTTSSPR--LF
FG10911.1 	MIP-QLLQRSSRACPRYNPALYRLSTTSQQRPGLTQTF-------WTSAPRREQPR--TP
AN5528.1  	MLRQAAQTRCWRTPARFVSHRPAFPRSNPVSAALGGTQFRKLSVKTQTDKNTLSDKKTAE
          	*  .           *               . :          .  :       :.:  

MG03406.1 	APPTPPSSSTGPGSPRSPAVTPS----KDFLATPDKTNQEQRKADWAIIKEMSHYLWPKD
NCU05029.1	APNGSAKDESKPAVSTVPKTTGRG--PSDPLAAIDKTAQEQRKADWAIMKEMSKYLWPKG
FG10911.1 	TDSKPTTTKPSAVFPANKKATKQ----NDPLVTVEKSAPEQRKADWAIMKEMTRYLWPKD
AN5528.1  	ADTSSSSSSAKSAAPSQQKTQNATTAQKNLLSESTVANKEQRKADWAIMREMAKYLWP--
          	:   ... .. .  .    .    :: .: *     :  *********::**::****  

MG03406.1 	SFNTKMRVALAVSLLISAK~VLNVQVPFYFKSIVDAMNVDVGAMGGTAAAVAGSMILAY~
NCU05029.1	SWGDKARVLLAIGLLVGGK~VLNVQVPFYFREIVDSLNIDFSTTGGSVTAVAGAMILGY~
FG10911.1 	DWGTKLRVSLAVSLLIGAK~VLNVQVPFYFKSIVDSMNIDVAAVGGTATTVAGAMILAY~
AN5528.1  	------------------K0VLNVEVPFYFKSIVDSMNVDFAAVGGTAYTVAGSMIIAY1
          	                  * ****:*****:.***::*:*..: **:. :***:**:.* 

MG03406.1 	GATRIGATVFQELRNAVFASVAQKAIRKVAANVFDHLLRLDLAFHLSKQTGGLTRAIDRG
NCU05029.1	GAARVGAVVSQELRNAVFASVAQKAIRKVARNTFEHLLNLDLSFHLSKQTGGLTRAIDRG
FG10911.1 	GASRIGATVFQELRNAVFASVAQNAIRKVACNVFDHLLRLDLTFHLSKQTGGLTRALDRG
AN5528.1  	GATRIGATFFQELRNAVFASVAQKAIRKVARNVFEHLLRLDLNFHLSRQTGGLTRAIDRG
          	**:*:**.. *************:****** *.*:***.*** ****:********:***

MG03406.1 	TKGISFLLTSMVFHVIPTALEITMVCGIL~TWQYGAQFAMITAVTMSAYTAFTIWTTAWR
NCU05029.1	TKGISFLLTSMVFHIVPTALEISMVCGIL~TYNFGWQYAALTALTMVSYTAFTILTTAWR
FG10911.1 	TKGISFILSSMVFHVLPTALEISMVCGIL~TYNYGAKFAALTVLTMVSYTAFTIWTTAWR
AN5528.1  	TKGISFLLTSMVFHVVPTALEISLVCGIL0THQYGIKFAAITATTMLAYSAFTIATTAWR
          	******:*:*****::******::***** * ::* ::* :*. ** :*:**** *****

MG03406.1 	TKFRRQANAADNRASTVAVDSLINYEAVKYFNNEKYEVKRYDDALKQYEKSSIKVATSLA
NCU05029.1	TKFRRQANAADNKASTIAVDSLINYEAVKYFNNEAYEVGRYDKALAQYEKNSIKVATSLA
FG10911.1 	TKFRRQANAADNKASTVAVDSLINYEAVKYFNNEKFEVARYDKALGQYEKNSIKVATSLA
AN5528.1  	TKFRKQANAADNRGATVAVDSLINYEAVKYFNNEKFEVARYDKALKAYEDASIKVTTSLA
          	****:*******:.:*:***************** :** ***.**  **. ****:****

MG03406.1 	FLNSGQNIIFSSALTAMMYLGANGIAQGNLSVGDLVMINQLVFQLSVPLNFLGSVYRELR
NCU05029.1	FLNSGQNIIFSSALTVMMYMGAHGVATGQLTVGDLVLINQLVFQLSVPLNFLGSVYRELR
FG10911.1 	LLNSGQNIIFSSALTGMMYLAANGVAEGTLTVGDLVMVNQLVFQLSVPLNFLGSVYRELR
AN5528.1  	FLNSGQNMIFSSALAAMMYLAADGVATGSLTVGDLVMVNQLVFQLSVPLNFLGSVYRELR
          	:******:******: ***:.*.*:* * *:*****::**********************

MG03406.1 	QSLLDMETLFNLQKVNVTIKEQPDAKPLSLSKGGEIRFENVNFGYHPDRPILRDLTLTIP
NCU05029.1	QSLLDMETLFNLQKVNVTIKEQPNAKPLTLTRGGEIEFKDVTFGYHPESPILRDLSLTIP
FG10911.1 	QSLLDMETLFNLQKVNANIKEKAGAKPLVLSKGGEIKFENVNFAYHPNRPILRDLSLTIP
AN5528.1  	QSLLDMETLFNLQKVNVNIKEKPDAKPLELKQGGQIRFENVTFGYHPERPILKNASFTIP
          	****************..***:..**** *.:**:*.*::*.*.***: ***:: ::***

MG03406.1 	AGKKVAIVGPSGCGKSTLLRLLFRFYDAQSGRILIDDQDIRSVQLDSLRKSIGVVPQDTP
NCU05029.1	AGKKVAIVGPSGCGKSTLLRLLFRFYDPQKGAIYIDGQDIRSVTLESLRRAIGVVPQDTP
FG10911.1 	AGKKVAVVGPSGCGKSTLLRLLFRSYDVQGGRVLIDDQDIRDVDLESLRRSIGVVPQDTP
AN5528.1  	AGQKFAIVGPSGCGKSTILRLLFRYYDVQEGRILVDGQDIRHVTIESLRKAIGVVPQDTP
          	**:*.*:**********:****** ** * * : :*.**** * ::***::*********

MG03406.1 	LFNETVELNIRYGDMSAPREQVVAAAKRARIHDTVESWPDGYATKVGERGLMISGGEKQR
NCU05029.1	LFNDTVEHNIRYGNLSATPEQVIEAAKAAHIHEKIISWRDGYNTKVGERGLMISGGEKQR
FG10911.1 	LFNDTVEHNIRYGSINATHEEVVAVAKRAHVHNTIQSFPDGYETKVGERGLMISGGEKQR
AN5528.1  	LFNDTIEHNIRYGRLDASDEEVRKAARRAHIHELVERLPEGYRTAVGERGMMISGGEKQR
          	***:*:* ***** :.*. *:*  .*: *::*: :    :** * *****:*********

MG03406.1 	LAVSRLLLKDPPLLFFDEA~TSALDTHTEQALMQNINSILR---EKS--RTSVFVAHRLR
NCU05029.1	LAVSRLILKDPPLLFFDEA~TSALDTHTEQALMENINAILKGLGQKGEKKTSLFVAHRLR
FG10911.1 	LAVSRILLKDPPLLFFDEA~TSALDTHTEQALMMNINSILR---ERG--RTSVFVAHRLR
AN5528.1  	LAISRLLLKDPQLLFFDEA0TSALDTYTEQALMQNINSILK---EKG--RTSVFVAHRLR
          	**:**::**** ******* ******:****** ***:**:   ::.  :**:*******

MG03406.1 	TIYDSDLIIVLKEGHVAEMGSHKELIDRGGLYSELWSA1QETLFNADGSEKNEE--VPDK
NCU05029.1	TIYDSDLIIVLKEGRVAEQGTHRELMERNGVYAQLWRA~QEMLMTEEGEVSKKG--EKEE
FG10911.1 	TIFDSDLIIVLKEGHVAEMGTHRELIDRDGVYAELWSA1QETMFGEDGKEKSEE--ENDE
AN5528.1  	TIYDCDQILVLKDGQVAELGSHRELLDLDGIYAELWSA1QETSLAQDPEYERNAGLEGET
          	**:*.* *:***:*:*** *:*:**:: .*:*::** * **  :  : . . : .   : 

MG03406.1 	SGRPRS-----
NCU05029.1	VGEKKEA----
FG10911.1 	QKKN-------
AN5528.1  	AGEVEDKAPRQ
          	  .  . :. .
```
